# Langerhans Cell Histiocytosis of the Cranial Base: Is Low-Dose Radiotherapy Effective?

**DOI:** 10.1155/2012/789640

**Published:** 2012-08-16

**Authors:** Andreas Meyer, Michael Stark, Johann H. Karstens, Hans Christiansen, Frank Bruns

**Affiliations:** Department of Radiation Oncology, Hannover Medical School, Carl-Neuberg-Street 1, 30625 Hannover, Germany

## Abstract

*Introduction*. Langerhans cell histiocytosis (LCH) is a rare disease of unknown etiology with different clinical features. A standardised treatment has not been established so far. *Case Report*. We report a case of a 28-year-old patient who initially presented with hypesthesia of the fifth cranial nerve and pain of the left ear. Diagnosis showed a tumour localised in the cranial base with a maximum diameter of 4.1 cm. The diagnosis of LCH was confirmed histologically by biopsy. Diagnostic workup verified the cranial lesion as the sole manifestation of LCH. A total dose of 9 Gy (single dose 1.8 Gy) was delivered. The symptoms dissolved completely within 6 months after radiation; repeated CT and MRI scans revealed a reduction in size of the lesion and a remineralisation of the bone. After a followup of 13 years the patient remains free of symptoms without relapse or any side effects from therapy. *Discussion*. Due to the indolent course of the disease with a high rate of spontaneous remissions the choice of treatment strongly depends on the individual clinical situation. In the presented case low-dose radiotherapy was sufficient to obtain long-term local control in a region with critical structures and tissues.

## 1. Introduction

Langerhans cell histiocytosis (LCH) is a rare and benign chronic inflammatory disease of unknown aetiology characterised by idiopathic proliferation of histiocytes with different clinical features mostly occurring in children [1]. The name LCH includes some synonyms formerly used for the description of different clinical pictures based on the local or disseminated proliferation of histiocytes [2]. The extent in severity of disease can range from systemic disease to an isolated lesion with the cranial base and the proximal femur being the most common sites of osseous LCH [3]. In case of involvement of the central nervous system diabetes insipidus is a symptom that can be found [4]. The optimal therapy strongly depends on the individual clinical situation of the patient. We present a case report of a patient with localised LCH of the cranial base treated by low-dose radiotherapy.

## 2. Case Report

**Figure 1:**
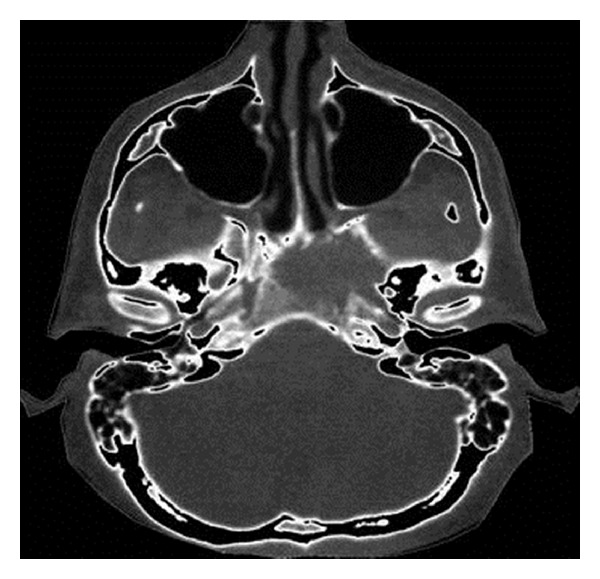
Cranial CT before radiotherapy.

**Figure 2:**
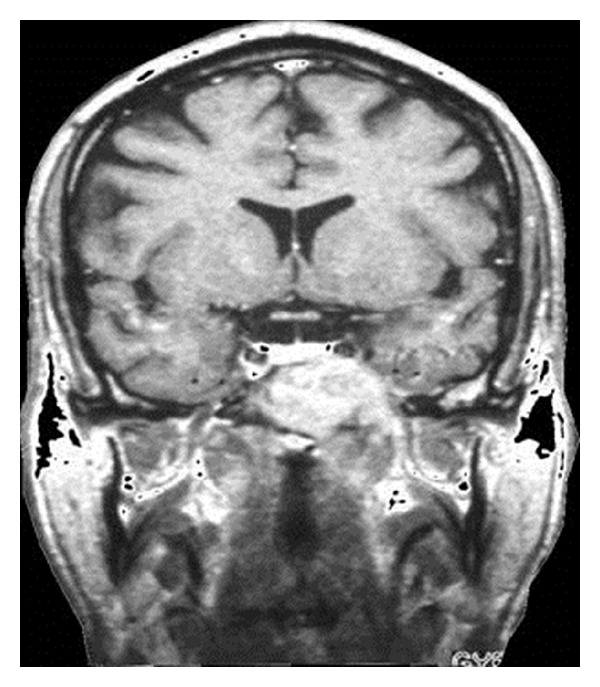
Cranial MRI before radiotherapy.

**Figure 3:**
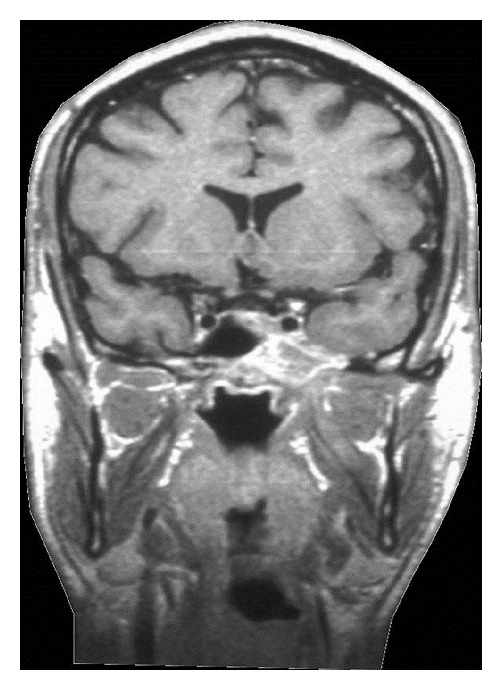
Cranial MRI 5 weeks after radiotherapy.

**Figure 4:**
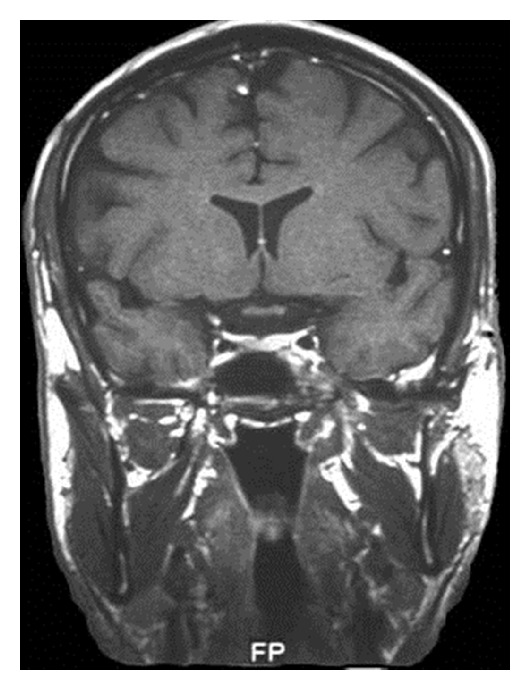
Cranial MRI 16 months after radiotherapy.

A 28-year-old patient initially presented with hypesthesia of the left-sided fifth cranial nerve and with pain of the left ear and eye lasting for approximately 5 weeks. Diagnostic workup including CT and MRI scans of the head showed a tumour localised in the left-sided cranial base with a maximum diameter of 4.1 cm with destruction of the Foramina lacerum and ovale and the dorsal wall of the sphenoidal sinus (Figures 1 and 2). The differential diagnosis included LCH, lymphoepithelioma, and nasopharyngeal cancer. Further diagnostic evaluation including CT of the chest and abdomen, ultrasound of the abdomen, and scintigraphy of the skeletal system verified the cranial lesion as the sole manifestation. Diagnosis was confirmed histologically by fine-needle biopsy of the sphenoidal sinus revealing histiocytosis (eosinophilic granuloma) of the cranial base. Due to the involvement of a cranial nerve with neurologic symptoms the indication for radiotherapy was given. A total dose of 9 Gy to the cranial base was delivered with 11 × 8.5 cm fields in 5 fractions with a single dose of 1.8 Gy via lateral opposed fields using 6 MV photons of a linear accelerator. One month after RT the pain and the hypesthesia improved; 6 months after RT the symptoms had dissolved completely. Repeated CT and MRI scans of the head 5 weeks and 6 months after the radiotherapy revealed a reduction in size of the lesion and a remineralisation of the bone (Figure 3). MRI scans of the head during further followup showed no evidence of disease (Figure 4). After a followup of 13 years the patient remains free of symptoms without relapse or any side effects from therapy.

## 3. Discussion

The associated symptoms of LCH depend on the location of the disease; the prognosis is related to the number of involved organs [3, 5]. Patients with bone lesions in which minimal therapy is often sufficient for long-time control have the best prognosis in comparison with patients with involvement of other organ systems [3, 6, 7]. Localized forms of LCH typically affect a single or several bones with the skull being mostly involved [8]. However, also in primary localised disease there is a risk of further dissemination. A bioptic verification of the disease and an adequate staging should be carried out in all patients to establish the correct diagnosis and to initiate the therapy [9].

However, due to the small number of patients and the heterogeneous picture of this disease a standardised treatment has not been established so far. Additionally, due to the indolent course of the disease with a high rate of spontaneous remissions the choice of treatment strongly depends on the individual clinical situation. Beside a wait-and-see policy surgery, radiotherapy, chemotherapy or combination of these therapy modalities is used [9]. In case of asymptomatic disease a close-clinical and radiographic followup can be carried out [10]. Surgery or radiotherapy is often used for localised disease whereas chemotherapy is the treatment of choice in disseminated disease. 

In localised disease radiotherapy can be useful in relieving pain and symptoms on the one hand and preventing from fracture of bone lesions on the other hand, either as a single treatment modality or in combination [7]. Radiotherapy of LCH is a highly effective therapeutic option with only minimal side effects preserving the function of the organ involved if complete surgical resection is connected with loss of function or if a complete surgical resection is not possible [6, 11, 12]. However, the dose-effect relationship still is a matter of debate [3, 6, 7]. In the literature single doses of 1.5 to 2 Gy and total doses up to 40 Gy have been recommended [6, 7, 9, 11–14]. However, a total dose of 9–12 Gy has proven to be effective achieving a local control rate of approximately 90% [6, 7, 11–13, 15]. In the presented case low-dose radiotherapy with a total dose of 9 Gy was sufficient to obtain long-term local control. Higher doses should be avoided due to the risk of induction of late toxicities and secondary malignancies in mostly young patients [3]. Additionally in case of relapse of disease irradiation can be delivered again without the risk of enhanced late effects.

In conclusion, low-dose radiotherapy for a solitary bone lesion is sufficient to obtain long-time control with preserving the function of the involved organ or tissue. Higher doses >20 Gy should be avoided because no dose-effect-relationship is described in this disease.
